# Evolving Chemotherapy Free Regimens for Acute Promyelocytic Leukemia

**DOI:** 10.3389/fonc.2021.621566

**Published:** 2021-02-25

**Authors:** Uday Kulkarni, Vikram Mathews

**Affiliations:** Department of Haematology, Christian Medical College & Hospital, Vellore, India

**Keywords:** differentiation therapy, arsenic trioxide, acute promyelocytic leukemia, all-trans retinoid acid (ATRA), non-chemotherapeutic treatment

## Abstract

With the treatment advances over the last three decades, acute promyelocytic leukemia (APL) has evolved from being the most malignant form of acute leukemia to a leukemia with excellent long term survival rates. In the present review, we have summarized data leading to the development of the currently used treatment regimens for APL, which incorporate either none or minimal chemotherapeutic drugs. We have discussed the historical aspects of APL treatment along with the challenges associated with chemotherapy-based approaches and our experience with the use of single agent arsenic trioxide (ATO) which was one of the first successful, non-chemotherapy approaches used for APL. Subsequently, we have reviewed the data from major clinical trials in low-intermediate risk APL and high risk APL which guide the current clinical practice in APL management. With accumulating data on oral ATO, we postulate that the treatment for low-intermediate risk APL will be a completely oral ATO + ATRA regimen in the future. While for high-risk APL, we believe that minimal anthracycline use with ATO + ATRA might become the standard of care soon. A number of promising non-chemotherapy drugs with pre-clinical data would merit clinical testing in the high risk and relapsed setting, with potential to translate to a complete oral chemotherapy free combination regimen in combination with ATO and ATRA.

## Introduction

Acute promyelocytic leukemia (APL) is a distinct form of acute myeloid leukemia (AML) characterized by t(15;17), a reciprocal translocation leading to a fusion transcript *PML-RARA.* This leads to a block in differentiation of the leukemic cells at the stage of promyelocytes.

APL was first described in 1957 by a Norwegian physician in a case series involving three patients, all of whom were treated with steroids and died. It was noted that APL has a very rapid fatal course and has a severe bleeding tendency making it the “most malignant form of acute leukemia” (1). Subsequently, daunorubicin was noted to result in remission in almost 70% patients with median remission duration of about 2 years. The responses and survival rates, at those times, were similar to those in other patients with a diagnosis of AML (2, 3). However, unique to this sub-type of AML was the exacerbation of a coagulopathy, associated with fatality in a proportion of cases, after initiation of chemotherapy (4).

### All-Trans Retinoic Acid

The advent of all-trans retinoic acid (ATRA) has revolutionized the treatment in APL with high complete remission (CR) rates without myelosuppression, reduced length of hospitalization, dramatic reduction in coagulopathy, reduced transfusion requirements, and reduction in early treatment related mortality (5). However, despite high CR rates, none of the patients were cured with single agent ATRA (6). It was subsequently demonstrated through a series of landmark studies that a combination of chemotherapy and ATRA used upfront during induction, along with a risk-adapted consolidation approach resulted in excellent long-term survival rates exceeding 80% (7–11).

### Arsenic Trioxide

The single most potent therapy for APL both in relapsed as well as upfront therapy for APL is arsenic trioxide (ATO) (12, 13). Historically, arsenic compounds have been used for treating various ailments, like chronic myeloid leukemia, trypanosomiasis, and dermatological conditions including syphilis (14). In China, at the Harbin Medical University, “Ai ling No 1”, an arsenic-based traditional Chinese recipe, was evaluated methodically for its therapeutic role in various malignancies (15). This preparation was called 713 (for the year and month of the initiation of this study). They studied more than 1,000 patients with various malignancies and noted that this preparation was maximally beneficial for the treatment of patients with APL. Subsequent studies also confirmed these observations (4). Single agent ATO was shown to result not only in excellent CR rates but also that these remissions were durable, in patients with APL relapsing after ATRA+chemotherapy (12). The dose used was 10 mg daily for adults till achievement of CR. This dose was derived from experience with the doses used in Chinese native medicines and not formal Phase I clinical trials. Subsequently, it was found that ATO was active in APL in doses ranging from 0.06 mg/kg to 0.2 mg/kg and that a dose of 0.15 mg/kg/day can be used in children (16). Various strategies for treating APL incorporating ATO include: upfront use of single agent ATO, use of ATO after achieving remission with ATRA+chemotherapy, use of ATO+ATRA induction followed by chemotherapy consolidation, and use of ATO+ATRA and chemotherapy in induction (17). The long-term cure rates with a combination of ATO and ATRA in low-intermediate risk APL have exceeded 90%–95%, and hence this forms the current standard of care for treating low-intermediate risk APL (18, 19).

### Need for Non-Chemotherapy Approaches

Elimination of cytotoxic chemotherapy has potential advantages of reduction in myelosuppression and resulting infections and bleeding, reduction in early risk of hemorrhagic events partly attributed to release of procoagulants after destruction of APL cells, and reduction in risk of long-term complications like cardiotoxicity and secondary myeloid neoplasms (17). Additionally, the health resource utilization and treatment costs are also significantly lower with non-chemotherapy approaches in APL (20). This is of particular significance for low-middle income countries wherein the resources are limited and there is an increasing burden of antimicrobial drug resistance.

## Our Early Experience With Non-Chemotherapy Regimen–Single Agent ATO

At our institution, two patients with APL who relapsed following chemotherapy (one of them also received ATRA) were advised palliative care. They subsequently chose to take alternative medications containing arsenic (Ayurvedic preparation from Vaidya Balendu Prakash at Dehradun, India) and went into durable remissions. They were administered this preparation continuously and in one case, this was continued beyond 5 years. Of these, one was noted to have severe arsenic keratosis and subsequently died of secondary squamous cell carcinoma (4, 21).

Following this, treatment with single agent ATO for APL was evaluated in the setting of a clinical trial since 1998 at our center. Our hospital pharmacy manufactured the intravenous ATO in-house with appropriate quality control measures. This manufacturing process was subsequently transferred to the industry in the year 2001 (Intas Pharmaceuticals Ltd, Matoda, Gujarat, India). We demonstrated that single agent ATO led to durable remissions in newly diagnosed APL with 5-year overall survival of 74.2 ± 5.2%. The toxicity profile was acceptable with mild reversible toxicity in the majority. There were no sudden cardiac deaths or acute hepatic failure. There were no long-term toxicities in terms of cardiac dysfunction or second malignancies. Additionally, seven patients in our cohort attained normal parenthood after completing ATO treatment (22–24).

In addition to efficacy and safety data, we also reported the pattern of leukocyte recovery following single agent ATO therapy in APL. With ATO treatment, about 1/3^rd^ patients have initial prolonged leukopenia followed by gradual normalization of the counts while the remaining 2/3^rd^ patients have an initial leukocytic response. We used hydroxyurea (or anthracycline if WBC counts increased despite hydroxyurea) to manage the leukocytosis. The leukocytosis is usually followed by a leukopenic phase of variable duration following which there is recovery to normal WBC counts (21). We reported, for the first time, that FLT3 activating mutations and secondary cytogenetic changes were not associated with an adverse impact on clinical outcome in APL treated with ATO (23, 25).

We also studied the arsenic levels in hair and nail samples of patients and control subjects. We noted no significant difference in the arsenic retention between controls and patients who had completed treatment at least 2 years ago. Also, immediately at the end of treatment, the arsenic levels were less than the lower limit of the normal range defined by the Agency for Toxic Substances and Disease Registry (ATSDR), Atlanta, Georgia (24).

## Various Strategies of Using ATO in APL

Upfront use of single agent ATO has been shown to result in excellent long term survival rates in patients having WBC counts less than 5 × 10^9^/L and platelet count >20 × 10^9^/L, whereas 5-year overall survival for the high risk patients was 63% (24). Also multiple courses of ATO consolidation after single agent ATO induction has been shown to improve the disease-free survival in APL (26).

In the North American Leukemia Intergroup study C9710, addition of ATO in consolidation following ATRA + chemotherapy induction, resulted in improved event-free survival (80% versus 63% at 3 years, p 0.001) however the overall survival was similar (86% versus 81% at 3 years, p 0.059) (27).

Pre-clinical studies showed synergy between ATO and ATRA both in *in vitro* experiments with APL cell lines as well as in animal model experiments with a possible benefit of sequential therapy over concurrent treatment. These have further formed the rationale for early clinical studies using the chemotherapy free combination of ATO and ATRA (28, 29).

A combination of ATO and ATRA used during induction followed by chemotherapy-based consolidation has been shown to be better than either agent used alone in terms of the time to CR, time to platelet recovery, fold change in the *PML-RARA* transcripts and also the long-term relapse risk (30).

Use of ATO-ATRA along with idarubicin during induction followed by ATO+ATRA consolidation and maintenance with ATRA/6-Mercaptopurine/Methotrexate resulted in excellent long-term survival exceeding 90% in the Australian APML4 study, with reduction in anthracycline exposure to a large extent (31).

## Low and Intermediate Risk APL

Low-intermediate risk APL is defined as APL with initial white blood cell count ≤10 × 10^9^/L) (32). Randomized clinical trials (GIMEMA-AMLSG-SAL APL0406 and UK NCRI AML17) have demonstrated the superiority of combination therapy with ATO and ATRA (ATO + ATRA) over conventional therapy with ATRA and chemotherapy (ATRA + chemotherapy) in low-intermediate risk APL (18, 19).

### APL0406 (ClinicalTrials.gov Identifier: NCT00482833)

In the APL0406 trial, with the median follow-up being 40.6 months, treatment with ATO + ATRA resulted in significant improvement in the overall survival in patients with low-intermediate risk APL as compared to ATRA + chemotherapy treatment (99.2% versus 92.6% at 50 months, p = 0.0073). The updated results from the APL0406 study showed an increasing benefit over time with ATO + ATRA as compared to ATRA + chemotherapy. At a median follow up of 66.4 months, the 6-year event-free survival was significantly better with ATO + ATRA as compared to ATRA + chemotherapy (96.6% versus 77.4%, p 0.0002). The cumulative incidence of relapse was significantly lower with ATO + ATRA as compared to ATRA + chemotherapy (1.7% versus 15.5%, p 0.02).

The proportion of patients with grade 3–4 thrombocytopenia and neutropenia for more than 15 days, those with infections and fever of unknown origin, and those with grade 3–4 gastrointestinal toxicity was lower with ATO + ATRA as compared to ATRA + chemotherapy. The proportion of patients with grade 3–4 hepatotoxicity and QTc prolongation during induction, and those with all grades of neurotoxicity during consolidation was greater with ATO + ATRA as compared to ATRA + chemotherapy. The toxicity resolved in all patients with temporary discontinuation of therapy except one patient who required ATO’s permanent discontinuation. During induction, there were four deaths in the ATRA + chemotherapy group (1 cardiovascular, 1 ARDS, 1 ischemic stroke, and 1 respiratory disease) with none in the ATO + ATRA group. Post-induction, there were five deaths while in remission in the ATRA + chemotherapy group, of which two had therapy-related myeloid neoplasm, while two died while in remission in the ATO + ATRA group of which one had colon carcinoma. Additionally, there were four deaths due to relapse in the ATRA + chemotherapy group (19, 33).

### UK NCRI AML17 (International Standard Randomized Controlled Trial Number ISRCTN55675535)

The UK NCRI AML17 trial primarily looked at the quality of life measured using the European Organization for Research and Treatment of Cancer (EORTC) QLQ-C30 global health status in APL patients treated with ATO + ATRA versus ATRA + chemotherapy. They had used an attenuated ATO schedule with ATO 0.3 mg/kg IV for 5 days in week 1 followed by 0.25 mg/kg IV twice a week from week 2 onward during both induction and consolidation in variance with the daily ATO used in APL0406 trial (**Table 1**). They did not report a significant difference in the quality of life between the patients treated in the two groups studied. In fact, for cognitive and role functioning domains, significant benefits were recorded in favor of ATO + ATRA. Most patients (93%) with high risk APL in the ATO + ATRA group received gemtuzumab ozogamicin. In the updated results from the AML17 trial, at a median follow-up of 67.4 months, frank relapses were significantly lower with ATO (1% versus 5% at 5 years, p 0.005) resulting in improved relapse-free survival irrespective of the risk group (for low risk 95% versus 87%, p 0.3, while for high risk 100% versus 83%, p 0.03).

**Table 1:** Different treatment schedules and survival rates for major trials on APL.

Study	Induction	Consolidation/ Maintenance	Toxicity	Survival data	Cumulative ATO and anthracycline dose
APL0406 (low-intermediate risk) (19, 33) ATO+ATRA arm	ATO 0.15mg/kg IV + ATRA 45mg/m^2^ PO – maximum of 60 days (median time to CR – 32 days)	ATO 0.15mg/kg IV for 5 days per week, 4 weeks on 4 weeks off, for 4 courses and ATRA 45mg/m^2^ PO daily 2 weeks on and 2 weeks off, for 7 courses	No induction deaths; 2 deaths while in remission post induction	At 50 months, OS of 99.2% (95%CI: 97.7 to 100)	16.8mg/kg to 21mg/kg ATO
APL0406 (low-intermediate risk) (19, 33) ATRA+CHT arm	Idarubicin 12mg/m^2^ IV on days 2,4, 6, and 8 along with daily ATRA 45mg//m^2^ PO for up to 60 days	Idarubicin 5mg/m2/day IV for days 1 to 4 (first cycle), mitoxantrone 10mg/m2/day IV on days 1-5 (second cycle) and idarubicin 12mg/m2 IV on day 1(third cycle). ATRA 45mg/m2/day PO from day 1 to day 15 during each consolidation cycle. Maintenance – 6-MP 50mg/m2/day, MTX 15mg/m2/week alternating with ATRA 45mg/m2/day given for 15 days every 3 months	4 deaths during induction and 5 while in remission post induction	At 50 months, OS of 92.6% (95%CI: 87.9 to 97.5)	80mg/m2 of idarubicin and 50mg/m2 of mitoxantrone
UK AML 17 (all risk groups) (18, 34) ATO+ATRA arm	ATO 0.3mg/kg IV for 5 days in week 1 followed by 0.25mg/kg twice a week for week 2 to 8.ATRA 45mg/m2 PO daily up to 60 days GO 6mg/m2 single dose within 4 days for high risk.	Course 2 to 5: ATO 0.3mg/kg IV for 5 days in week 1 followed by 0.25mg/kg twice a week for week 2 to 4. ATRA 45mg//m2 PO 2 weeks on and 2 weeks off.	60-day mortality – 5%, Therapy related myeloid neoplasms - none	5-year OS 92%	17mg/kg ATO
UK AML 17 (all risk groups) (18, 34) ATRA+CHT arm	Idarubicin 12mg/m2 IV on days 2,4, 6, and 8 along with daily ATRA 45mg/m2 PO for up to 60 days	Idarubicin 5mg/m2/day for days 1 to 4 (first cycle), mitoxantrone 10mg/m2/day on days 1-5 (second cycle) and idarubicin 12mg/m2 on day 1(third cycle). ATRA 45mg/m2/day from day 1 to day 15 during each consolidation cycle.	60-day mortality – 9%; Therapy related myeloid neoplasms – 6%	5-year OS 86%	80mg/m2 of idarubicin and 50mg/m2 of mitoxantrone
APML4 study (All risk groups) (35)	ATRA 45mg/ m2 days 1-36. Idarubicin 12mg/ m2 for age 1 to 60 years on day 2, 4, 6, and 8 ATO 0.15mg/kg IV days 9 to 36	ATRA 45mg/ m2 and ATO 0.15mg/kg IV from day 1 to 28 in cycle 1. ATRA 45mg/ m2 on days 1-7, 15-21, 29-35 and ATO 0.15mg/kg IV 5 days in a week for 5 weeks in cycle 2. 8 cycles of maintenance: ATRA 45mg/ m2 days 1-14 along with MTX 5-15mg/ m2 and 6-MP 50-90 mg/ m2 on days 15-90.	Early death rate - 3.2%; No therapy related myeloid neoplasm	5-year OS 96% (95%CI: 90-99) for low-intermediate risk and 87% (95%CI: 65-96) for high risk	12.15mg/kg ATO 48mg/m2 of idarubicin
MDACC (All risk groups) (36, 37)	ATO 0.15mg/kg IV + ATRA 45mg/m2 PO – till CR (median of 30 days) GO 9mg/m2 or Idarubicin 12mg/m2 on day 1 in case of high risk	ATO 0.15mg/kg IV 5 days per week, 4 weeks on 4 weeks off, for 4 courses and ATRA 45mg/m2 PO daily 2 weeks on and 2 weeks off for 7 courses	Induction mortality - 4%	5 year OS 89% for low risk and 86% for high risk	16.5mg/kg ATO 12mg/m2 idarubicin
SWOG 0535 (high risk) (38)	ATO 0.15mg/kg IV + ATRA 45mg/m2 PO – till CR (median of 39.5 days) GO 9mg/m2 on day 1	ATO 0.15mg/kg IV for 25 days (Cycle 1 and 2). Daunorubicin 50mg/m2 for 3 days and ATRA 45mg/m2 for 7 days (cycle 3 and 4) and GO 9mg/m2 on day 1 (cycle 5 and 6) Maintenance 1 year : ATRA 45mg/m2 for 7days (every 14 days) with 6-MP 60mg/m2 and MTX 20mg/m2 weekly	6-week mortality – 11%	3 year OS of 86% (95%CI: 75 to 92)	13.5mg/kg ATO 300mg/m2 daunorubicin

(APL, acute promyelocytic leukemia; CHT, chemotherapy; CR, complete remission; ATO, arsenic trioxide; ATRA, all-trans retinoic acid; GO, gemtuzumab ozogamicin; 6-MP, 6-mercaptopurine; MTX, methotrexate; OS, overall survival).

The 60-day mortality was similar with ATRA + chemotherapy and ATO + ATRA (9% versus 5%, p 0.22). The causes of death in the ATO + ATRA group were: 3 cardiac events, 1 renal failure, 1 infection, and 1 due to several causes, while for 11 patients in the ATRA + chemotherapy group were: 3 hemorrhages, 3 infections, 2 pulmonary causes, 1 renal cause, and 2 progressive disease. The incidence of therapy-related myeloid neoplasm was 6%, with no case seen after ATO + ATRA. Of 32 patients (including 17 with a molecular relapse) who relapsed on ATRA + chemotherapy group, 31 patients attained molecular CR with ATO and had 5 year survival of 88% with a median follow-up of 44.9 months. The highly effective salvage therapy with ATO and minimal residual disease monitoring resulted in a lack of overall survival benefit between the two arms of the AML17 trial in variance with the APL0406 trial. Hyperbilirubinemia, cardiac events, gastrointestinal events, and alopecia were more frequent in the ATRA + chemotherapy arm during treatment course 1. The proportion of patients with high alanine transaminase (ALT) levels between the two groups was similar. After treatment course 1, the grade 3–4 ALT elevation was more common with ATO + ATRA than ATRA + chemotherapy (20% grade 3 and 5% grade 4 versus 8% grade 3 and 2% grade 4). After course 2, liver toxicities did not differ between the 2 groups while cardiotoxicity was more frequent with ATO + ATRA (7% grade 1–2, 3% grade 3, and none grade 4). Treatment discontinuation was required for 2 patients on ATO+ATRA while on induction and for 6 while on consolidation (as per clinician’s decision, with 2 having QTc prolongation). ATO + ATRA was associated with lesser days of hospitalization, lower transfusion, and intravenous antibiotic requirement as compared to ATRA + chemotherapy during the first two courses of therapy (18, 34).

## High Risk APL

High risk APL is defined as APL with initial white blood cell count >10 × 10^9^/L) (32). In high-risk APL, broadly two approaches—either with minimal anthracycline use during induction or with the use of gemtuzumab ozogamicin, have been combined with ATO + ATRA.

### APML4 Study (Australian New Zealand Clinical Trials Registry, number ACTRN12605000070639)

The APML4 study was a non-randomized phase 2 trial which looked at the freedom from relapse and early death with the addition of ATO to ATRA and idarubicin in induction with an ATO + ATRA consolidation. Thus, there was minimal use of anthracycline limited to induction therapy alone. However after two cycles of consolidation, patients received maintenance therapy with ATRA, 6-mercaptopurine, and methotrexate for 2 years. The results were compared with that of the earlier APML3 study which had excluded ATO. The early death rate was lower (3.2% versus 7.1%) however this did not achieve statistical significance. Among the 124 patients studied, 23 were high risk as per the Sanz risk stratification. Among the high risk patients, on comparison with the APML3 results, the freedom from relapse was significantly better (2-year rate of 95% versus 69%; p 0.024) however the overall survival was similar (2-year OS 87% versus 87%; p 0.40). For high risk disease, the cumulative incidence of relapse was 5% at 5 years compared to 31% at 2 years in the APML3 study. Also there were no deaths in remission during consolidation. There were no instances of secondary myelodysplasia or leukemia reported (35).

### Gemtuzumab Ozogamicin

Addition of gemtuzumab ozogamicin (GO) to the combination of ATO + ATRA in induction is a promising approach which has been evaluated in multiple studies (36–38). In the MD Anderson Cancer Center (MDACC) trial (ClinicalTrials.gov Identifier: NCT01409161), of the 187 patients studied, 54 were high risk. The induction mortality was 4%. The CR rate in low risk as well as high risk patients was 96% with a median time for attaining CR of 30 (17–80) days. Among the 54 high risk patients, 45 received GO 9mg/m^2^ on day 1 while the remaining received idarubicin 12 mg/m^2^ on day 1 due to lack of availability of GO. The 5-year EFS and OS for the high risk group was 81% and 86% respectively. Five patients with high risk disease developed disease relapse. Treatment related adverse events included hepatotoxicity grade 3 and above in 14% patients and infections grade 3 and above in 23.5% patients.

Similarly, as mentioned earlier in the UK NCRI AML17 study GO was added to ATO+ATRA in the high risk subset and was associated with a 100% relapse-free survival, however the study was not powered to address efficacy in this subset.

In the SWOG study (ClinicalTrials.gov Identifier: NCT00551460), patients with high risk APL were treated with ATO + ATRA and GO in induction, followed by consolidation with two cycles of ATO, two cycles of ATRA with chemotherapy (daunorubicin) and two cycles of GO. They also included 1 year of maintenance with ATRA, 6-mercaptopurine, and methotrexate. They studied 70 patients with high risk APL. The 6-week mortality rate was 11% and 86% patients achieved CR at the end of induction. Treatment related adverse events during induction included about elevated liver enzymes grade 3 and above in 16% patients and infections grade 3 and above in 20%. The 3 year EFS was 78% (95% confidence interval: 67% to 86%). About 37% patients in remission were not able to complete the planned post-remission therapy including 12% due to adverse events.

## Oral ATO

Three oral formulations of ATO are being developed. One is a liquid formulation and has been shown to have comparable efficacy similar to IV ATO. The safety and efficacy of this formulation has been established in frontline setting given after ATRA and chemotherapy (39). The second one is oral arsenic realgar-Indigo naturalis formula (RIF) given as 60 mg/kg bodyweight daily. In non-high risk patients, this formulation is non-inferior to intravenous ATO with a similar adverse effect profile (40). Thirdly, ORH-2014 is another oral formulation for which pharmacokinetic and safety data is available (41).

## Other Potential Non-Chemotherapy Options in APL

### Targeting Microenvironment Mediated Resistance to ATO by Downregulating the NK- kB Pathway

**Figure 1:**
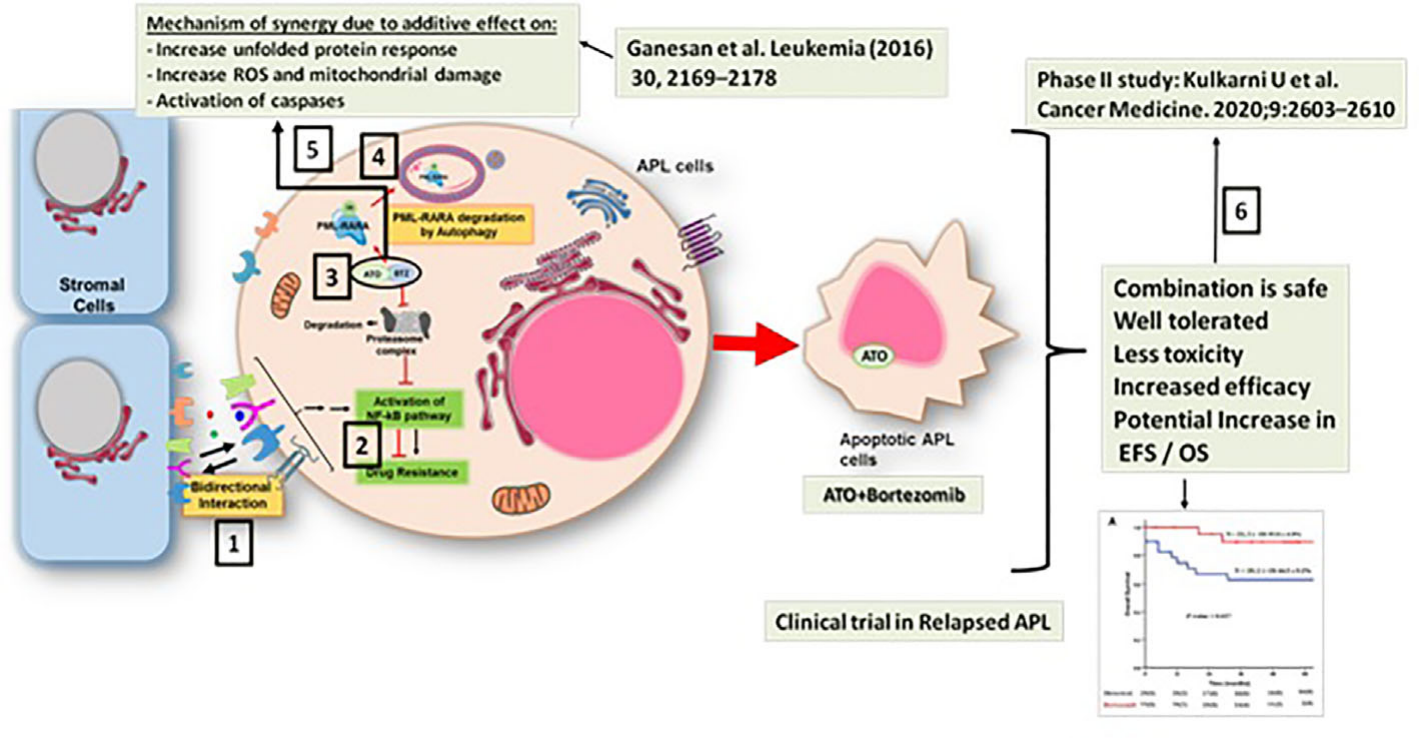
Synergy between arsenic trioxide (ATO) and bortezomib in the treatment of acute promyelocytic leukemia. 1. There is a significant contribution of microenvironment mediated resistance to ATO at relapse (41). 2. This resistance is mediated predominantly by the NF-kB pathway (41). 3. Preclinical studies show a synergistic effect of bortezomib and ATO in overcoming ATO resistance (42). 4. With the combination of ATO and bortezomib, the PML-RARA oncoprotein is cleared through a p62-dependent autophagy pathway (42). 5. The mechanism of the synergy between ATO and bortezomib involved downregulation of the NF-kB pathway, increased unfolded protein response, and an increased generation of reactive oxygen species in the malignant cell (42). 6. This work has been translated to a phase 2 clinical trial for relapsed APL wherein we have demonstrated that the combination of ATO, ATRA, and anthracycline with the addition of bortezomib is safe (43).

We have been using ATO-based therapy for upfront treatment of APL for the last two decades (24). We have shown that there is a significant contribution of microenvironment mediated resistance to ATO at relapse (42) and demonstrated the importance of the NF-kB pathway in mediating this resistance. We further demonstrated a synergistic effect of bortezomib, a proteasome inhibitor which is known to downregulate the NF-kB pathway, and ATO in overcoming ATO resistance in pre-clinical studies. This synergy is due to downregulation of the NF-kB pathway, increased generation of reactive oxygen species in malignant cells, and an increased unfolded protein response. Since ATO is known to cause PML-RARA degradation through the proteasome, a theoretical concern with the use of a proteasome inhibitor with ATO was that whether the proteasome inhibitor would inhibit the degradation of the PML-RARA oncoprotein. However, we noted that with the combination of ATO and bortezomib, the PML-RARA oncoprotein is cleared through an alternative p62-dependent autophagy pathway (43). This work has been translated to a phase 2 clinical trial for relapsed APL wherein we have demonstrated that a combination of ATO, ATRA, and anthracycline with the addition of bortezomib is safe (44) (**Figure 1**).

### Targeting Epigenetic Resistance to ATRA by Activating MEK/ERK Signaling

Drug resistance to ATRA is mediated by mutations in the ligand binding domain of PML-RARA or epigenetic modifications in the RARA promoter preventing expression of genes targeted by retinoic acid. The permissiveness of the RAR promoter is restored by activating the MEK/ERK signaling pathway (45).

It has also been demonstrated that the *in vitro* resistance to ATRA in APL cell lines can be overcome by use of glycogen synthase kinase-3beta inhibitors including lithium chloride by restoring ATRA-induced differentiation. This effect is abolished by inhibition of the MEK/ERK1/2 pathway. In *in vivo* mouse models, lithium chloride combined with ATRA resulted in a significant survival advantage as compared to ATRA alone (46).

### Targeting Energy Metabolism and Anti-Apoptotic Pathways

We have previously shown that there exists a significant change in the energy metabolism pathways in APL cell lines resistant to ATO (47). This forms a rationale for targeting APL with energy metabolism inhibitors.

The vitamin E derivative (+) α-tocopheryl succinate acts by inhibiting the mitochondrial respiratory chain complex I. It exerts pro-apoptotic effects in various tumors. In a mouse model of APL, this compound has been shown to be as effective as ATO or ATRA (48).

Also, targeting anti-apoptotic pathways can be potentially explored in APL. In fact, primary APL samples have been shown to be significantly more sensitive to venetoclax than non-APL AML samples (49). The therapeutic role of mitocans, such as venetoclax, in APL remains to be explored.

### Synergistically Enhancing Differentiation

Valproic acid also has been shown to induce differentiation in APL and has synergy with ATRA in preclinical models (50). Also, 1alpha, 25-dihydroxyvitamin D3 and vitamin K2 derivatives have been shown to augment retinoic acid induced differentiation of APL cell lines (51, 52).

## Way Forward

**Table 1** summarizes the major clinical trials in APL which have explored treatment approaches either without or with minimal chemotherapy. Non-chemotherapy approach consisting of ATO + ATRA is the current standard of care for treating low-intermediate risk APL based on evidence from randomized clinical trials. For high risk disease, the TUD-APOLLO-064 randomized controlled trial is evaluating ATO + ATRA + idarubin-based induction regimen for high risk APL patients with ATO + ATRA consolidation as post-remission strategy with event-free survival as the primary outcome and standard AIDA regimen as the comparator. The results of this study are expected in the near future and are eagerly awaited.

**Table 2** shows the anticipated treatment algorithm for APL in the future. With long term safety and efficacy data with oral ATO, we could possibly have a completely oral regimen of ATO + ATRA for low-intermediate risk APL. For high risk APL, if randomized data (TUD-APOLLO-064, ClinicalTrials.gov Identifier: NCT02688140) show superiority over conventional AIDA regimen, ATO + ATRA with minimal anthracycline use could be the standard therapy in the near future as shown in the anticipated treatment algorithm in **Table 2**. Other potential drugs that can be explored in the setting of high risk or relapsed disease have been shown in **Table 2**. Targeting microenvironment mediated drug resistance *via* the NF-kB signaling using bortezomib is an approach which we are currently exploring (43, 44). Combining glycogen synthase kinase-3beta inhibitors like lithium chloride with ATRA for targeting epigenetic mechanisms of resistance to ATRA *via* MEK/ERK signaling is another interesting approach. Also, lithium chloride is known to induce autophagy which is also a key component of ATRA induced differentiation in APL (46, 53). Targeting energy metabolism and antiapoptotic pathways using drugs like vitamin E and venetoclax respectively also could have potential therapeutic significance in high risk or relapsed APL, as could agents like vitamin D and Valproic acid showing synergy in differentiation in APL (48–51). All these approaches could pave the way for completely non-chemotherapy approaches in the high risk and relapse settings as well.

**Table 2:** Anticipated treatment algorithm for APL in future.

Treatment phase	Low-intermediate risk APL	High risk APL
Induction	Oral ATO + oral ATRA Dose and schedule optimization and possible addition of other non-chemotherapy agents to further reduce cumulative dose of ATO and ATRA and their side effects	Oral ATO + oral ATRA + either of the following: Minimal anthracycline, Gemtuzumab ozogamicin (targeting high CD33 expression on APL cells), Bortezomib (targeting microenvironment mediated ATO resistance *via* NF-kB signaling), potential for potent oral proteasome inhibitors to be evaluated here Glycogen synthase kinase -3beta inhibitors like lithium chloride (targeting epigenetic modification of the RARA promoter *via* MEK/ERK signaling), Vitamin E or venetoclax (targeting energy metabolism or anti-apoptotic pathways), Valproic acid or 1alpha, 25-dihydroxyvitamin D3 (synergistic differentiating activity with ATRA)
Consolidation	Oral ATO + oral ATRA	Oral ATO + oral ATRA

(APL, acute promyelocytic leukemia; ATO, arsenic trioxide; ATRA, all-trans retinoic acid).

Over the last few decades, APL has transformed from being the “most malignant form of acute leukemia” to the “most curable form of acute leukemia” (54). Other than development of non-chemotherapy approaches, the current controversies in this field include optimal management of early deaths related to coagulopathy (like the role of recombinant thrombomodulin), the optimal dosing of ATRA and ATO, the optimal therapy for high-risk APL, the role for intrathecal prophylaxis, the role of prophylactic corticosteroids during induction therapy, and the need for maintenance therapy after consolidation (55, 56). Limitations of the present review are that we have not covered these controversies in the management of APL other than the non-chemotherapy approaches and also that we did not have a pre-defined search strategy for literature review.

## Author Contributions

UK wrote the manuscript. VM conceptualized and reviewed the manuscript. All authors contributed to the article and approved the submitted version.

## Funding

Uday Kulkarni is supported by an early career fellowship program of DBT Wellcome India Alliance (IA/CPHE/17/1/503351), New Delhi, India. Vikram Mathews is supported by the senior fellowship program of the DBT Wellcome India Alliance (IA/CPHS/18/1/503930).

## Conflict of Interest

The authors declare that the research was conducted in the absence of any commercial or financial relationships that could be construed as a potential conflict of interest. 
